# Transcriptome Analysis of Effects of Folic Acid Supplement on Gene Expression in Liver of Broiler Chickens

**DOI:** 10.3389/fvets.2021.686609

**Published:** 2021-09-16

**Authors:** Yujie Zhang, Ningbo Zhang, Lin Liu, Yan Wang, Jinyi Xing, Xiuling Li

**Affiliations:** ^1^School of Life Sciences, Linyi University, Linyi, China; ^2^School of Agriculture and Forestry Sciences, Linyi University, Linyi, China; ^3^School of Pharmacy, Linyi University, Linyi, China

**Keywords:** RNA-seq, folic acid (FA), broiler, differentially expressed genes (DEGs), gene expression

## Abstract

Folic acid is a water-soluble B vitamin, and plays an important role in regulating gene expression and methylation. The liver is the major site of lipid biosynthesis in the chicken. Nevertheless, how gene expression and regulatory networks are affected by folic acid in liver of broilers are poorly understood. This paper conducted the RNA-seq technology on the liver of broilers under folic acid challenge investigation. First, 405 differentially expressed genes (DEGs), including 157 significantly upregulated and 248 downregulated, were detected between the control group (C) and the 5 mg folic acid group (M). Second, 68 upregulated DEGs and 142 downregulated DEGs were determined between C group and 10 mg folic acid group (H). Third, there were 165 upregulated genes and 179 downregulated genes between M and H groups. Of these DEGs, 903 DEGs were successfully annotated in the public databases. The functional classification based on GO and KEEGG showed that “general function prediction only” represented the largest functional classes, “cell cycle” (C vs. M; M vs. H), and “neuroactive ligand-receptor interaction” (C vs. H) were the highest unique sequences among three groups. SNP analysis indicated that numbers of C, M and H groups were 145,450, 146,131, and 123,004, respectively. Total new predicted alternative splicing events in C, M, and H groups were 9,521, 9,328, and 8,929, respectively. A protein-protein interaction (PPI) network was constructed, and the top 10 hub genes were evaluated among three groups. The results of real time PCR indicated that mRNA abundance of *PPAR*γ and *FAS* in abdominal fat of M and H groups were reduced compared with the C group (*P* < 0.05). Ultramicroscopy results showed that folic acid could reduce lipid droplets in livers from chickens. Finally, contents of LPL, PPARγ, and FAS in abdominal fat were decreased with the folic acid supplmented diets (*P* < 0.01). These findings reveal the effects of folic acid supplemention on gene expression in liver of broilers, which can provide information for understanding the molecular mechanisms of folic acid regulating liver lipid metabolism.

## Introduction

The consumption of chicken meat has increased rapidly in the past few decades due to its low cost and suitability for further processing (1, 2); consequently, chickens are one of the most reared species and the fastest growing animal product, especially in developing countries (2). However, the problem of excessive fat deposition in the body has increased with the development of intensive breeding, which has negative impacts on both feed efficiency and carcass quality for poultry production (3, 4). In particular, the abdominal fat pad was significantly heavier in fast-growing genotypes (such as Cobb) than slow-growing broiler in the modern poultry industry (5). In addition to genetic and environmental factors, nutritional level also plays an important role in lipid deposition in poultry. As one of the largest metabolic organs, liver plays an important role in nutrient metabolism, biosynthesis and immune defense. Especially, liver is the major site for lipid biosynthesis in the chickens (6, 7), and it is also the major site for folic acid (forms of which are known as folate) storage and processing (8). Folic acid is an important water-soluble vitamin that plays a role as a cofactor and a coenzyme in animal growth and development. Folic acid is taken up from the small intestine and then enters the liver through the portal vein. Folate metabolism involves at least 30 different enzymes in the liver, such as dihydrofolate reductase (DHFR), 5,10-methylenetetrahydrofolate reductase (5,10-MTHFR), tetrahydrofolic acid (THF) and so on (9, 10). The enzyme DHFR catalyzes the reduction of dietary folic acid or dihydrofolate to THF in the liver, and 5,10-MTHFR carries out a central reaction in folate metabolism (11). A study has shown that folic acid consumption has significant modulatory effects on lipolysis of obese/diabetic mice (12). Similarly, Kumar et al. reported that dietary restriction of folic acid and/or vitamin B_12_ in maternal and peri-/postnatal rats can increase visceral adiposity and alter metabolism in rat offspring (13). On the contrary, excessive maternal supplementation of folic acid in mice leads to an impairment in hepatic fat metabolism in the offspring (14).

To date, studies on the folic acid application in livestock and poultry diets have been increasing. In ewes, restricting the supply of folate and vitamin B_12_ before pregnancy leads to heavier and fatter adult offspring (15). *In vitro*, it was reported that folate increased the proliferation of adipocytes but reduced per-cell lipid accumulation in chickens (16). Also, folic acid addition could reduce lipid deposition in primary chicken hepatocytes (17). Recently, Liu et al. revealed that folic acid perfusion administration reduced abdominal fat deposition in broilers (18). On the other hand, it has been shown that the lipid and glucose metabolism of breeder cocks and broiler offspring were affected by paternal folic acid supplementation (19). Despite these findings, a comprehension of the effects of folic acid on the liver fat metabolism of chickens remains limited.

RNA-seq is a transcriptomics research method based on the second generation sequencing technology. It can accurately study cell transcriptomes with high resolution and depth. Our recent study of abdominal adipose in broiler offspring under maternal folate deficiency using digital gene expression profiling has found numerous differentially expressed genes (DEGs) involved in complex biologic processes including folate and lipid metabolic processes, methylation, and methyltransferase activity (20). In this work, we compare the effects of folic acid on transcriptomic profiles of broilers using RNA-Seq. Subsequently, bioinformatics analysis is carried out to systematically identify DEGs. Further analyses on gene ontology (GO), kyoto encyclopedia of genes and genomes (KEGG) pathway, single nucleotide polymorphism (SNP), new alternative splicing event and protein-protein interactions (PPI) are used to reveal up- and downregulated pathways in response to folic acid supplementation. In addition, real-time quantitative PCR (qPCR) analysis is performed to confirm the transcriptome results. Finally, lipid droplets in livers of chickens are assayed by transmission electron microscopy.

## Materials and Methods

### Ethics Statement

The experiment was conducted at Linyi University in China, and all animal experimental protocols were approved by the Animal Care and Use Committee.

### Animal and Diets

A total of 270 female broiler chicks (Arbor Acres) were supplied at 1-day-old by a commercial hatchery and were randomly allotted to three dietary treatment groups, each of which includes six replicate of 15 birds. Dietary treatment groups were: (1) basal diet + 0 mg/kg folic acid (control group, marked as C); (2) basal diet + 5 mg/kg folic acid (marked as M); (3) basal diet + 10 mg/kg folic acid (marked as H). Standard basal diet containing corn and soybean meal was fed as mash, and the diets were (Supplementary Table 1) formulated to provide all the nutrients to meet or exceed the nutrition requirements for broiler chickens by the National Research Council (NRC, 1994). The chicks were reared in floor pens with a stocking density of 0.09 cm^2^/bird. Water and diets were available *ad-libitum*, the lighting time was 24 h a day for the first week and then reduced to 16 hr during 8–42 days. The room temperature was 33°C during the first week and then decreased by 2°C every week until 25°C and the relative humidity was 60–70%.

### Slaughtering and Sampling

At the end of day 42, one bird around the average weight was randomly selected from each replicate group for sample collection after feed deprivation for 12 h. After slaughtering by bleeding from jugular vein, some liver and abdominal fat were collected. Then, the samples were isolated immediately, and washed briefly with PBS, snap-frozen in liquid nitrogen, and stored at −80°C for total RNA extraction. Meanwhile, other abdominal fat was also collected to assay contents of lipoprotein lipase (LPL), peroxisome proliferator-activated receptor γ (PPARγ) and fatty acid synthetase (FAS).

Tissues were also collected from different sites of each lobe of the liver for subsequent transmission electron microscopy.

### Library Construction and RNA-Sequencing

Total RNA was isolated from nine liver samples (25 mg per sample) (three replicate for each group) using Trizol Reagent (Invitrogen, Carlsbad, USA) according to the manufacturer's instructions, and then treated with DNase I to remove DNA contamination. The total RNA was characterized for quantitatively and qualitatively by Nanodrop ND-1000 spectrophotometer (NanoDrop Technologies) and 2100 Bioanalyzer (Agilent Technologyies) according to the manufacturer's instructions. Equal amounts of total RNA (at least 5 μg) were pooled from the three replicate within each group for cDNA library preparation.

RNA-seq libraries were generated using the NEBNext Ultra RNA Library Prep Kit for Illumina (NEB, Ipswich, MA, USA) following the manufacturer's instructions. Then transcriptome sequencing was performed on the Illumina HiSeq2500 platform (Illumina Inc., San Diego, CA, USA) that generated 100 bp paired-end raw reads.

### RNA-Seq Analysis

Raw data was filtered to remove adapters and low quality reads to obtain high-quality and cleaned data using fastx-toolkit tool. All cleaned data was mapped to the *Gallus gallus* genome (Gallus_gallus-5.0) using TopHat2 software (21) and Bowtie2 software (22). Based on the results of TopHat2 alignment between all cleaned reads and reference genome sequences, SAMtools software (23) was employed to identify the single base mismatch between all cleaned data and reference genome and search potential single nucleotide polymorphism (SNP) in gene regions. Only annotated SNPs with TopHat2 score ≥ 50, an interval of the single base mismatch site ≥ 5 bases, quality value of the variant recognition ≥ 20, and sequenced depth between 5 × and 100 × were accepted for downstream analyses. Alternative splicing events were predicted using Cufflinks2 version 2.2.1 (24). Cufflinks2 software was also employed to splice mapped reads and explore new transcripts or new genes. Then the new genes were aligned to public NR, Swiss-Prot, GO, COG and KEGG database using NCBI BLAST software.

Gene expression levels were characterized by FPKM values obtained using Cufflinks2 software. DEGs identified among groups were analyzed by the DESeq software (25). An adjusted fold change ≥2 and an FDR <0.01 were regarded as the threshold to determine DEGs. Additionally, the protein-protein interaction (PPI) network of DEGs was constructed by STRING v.11.0 (https://string-db.org/cgi/input.pl) with a minimum required interaction score of 0.4 (26). The PPI network was subsequently visualized using Cytoscape v.3.8.0 (27). The cyoHubba, a plug-in of Cytoscape, was used to screen the hub genes with the degree.

Hierarchical Clustering analysis of DEGs was conducted with Cluster 3.0 software. Gene annotation and GO terms were conducted using BLAST2GO (28). The database of Clusters of Orthologous Groups of proteins (COG) was used to predict and classify possible functions. Meanwhile, the DEGs were imported into the Cytoscape software (http://www.cytoscape.org/) with the ClueGO plugin (http://www.ici.upmc.fr/cluego/cluegoDownload.shtml) for KEGG pathway enrichment analysis. A Benjamini-corrected values of *P* < 0.05 were defined as significant.

### Quantitative Real-Time PCR

The residual RNA from same individual samples prepared for RNA-seq were also used for the qPCR verification to evaluate the accuracy of measurements of DEGs by RNA-seq. Moreover, RNA from abdominal fat was used to detected expression levels of *LPL, PPRA*γ and *FAS*. cDNA was synthesized using M-MLV RT (Promega Madison, WI, USA) following the manufacturer's instructions, and the primer sequences for these reactions are shown in Supplementary Table 2. The qPCR was carried out with the Brilliant SYBR Green qPCR Master Mix (Stratagene, La Jolla, CA, USA). Using the β*-actin* gene as the reference gene, the relative expression levels of samples were calculated by the 2^−Δ*ΔCT*^ method. All PCR reactions were performed in triplicate with negative controls.

### Transmission Electron Microscopy

Liver tissues were collected and cut into small pieces (<1 × 1 mm) and then immersed in 2% glutaraldehyde in phosphate buffer (pH 6.8) for 4 or 12 h at room temperature. After tissues were washed 6 times in distilled water to remove excess phosphate ions, a secondary fixation was carried out in 1% osmium tetroxide in phosphate buffer (pH 6.8) for 2 h at room temperature. Then dehydration was performed through a graded ethanol series. After replacing of ethanol by pure acetone, tissues were embedded in Embed-812 resin. Embedded tissues were sectioned at 60 nm thick on a Leica ultramicrotome. Sections were stained with 1% aqueous uranyl acetate for 10 min and then with lead citrate solution for 1 min at room temperature in dark. Observation and photography were performed under a Philips Tecnai 12 transmission electron microscope.

### Measurement of Contents of LPL, PPRAγ, and FAS in Abdominal Fat

Contents of LPL (Cat. No.: ml240584), PPRAγ (ml060856) and FAS (ml060836) in abdominal fat were analyzed using an enzyme-linked immunosorbent assay (ELISA) kit according to the manufacturer's guidelines (Shanghai Enzyme-linked Biotechnology Co., Ltd., Shanghai, China).

### Statistical Analysis

Data were analyzed with one-way ANOVA, followed by a Duncan's multiple range test using SPSS (Version 20.0, Inc., IL, USA). The GO and KEGG analysis was performed by Fisher's *t*-test. *P* < 0.05 was considered significant.

## Results

### Analysis of RNA-Seq Libraries

After removing the adaptors and filtering low quality reads from raw data, a total of 17.44 Gb cleaned data was obtained for three libraries of different supplemental folic acid in basal diet and the average cleaned data of per library were 4.77 Gb. The average Q30 value was higher than 89.05% (Supplementary Table 3). A total of 138,976,384 cleaned reads were generated for three groups. Meanwhile, 79.54–79.95% reads and 77.33–78.02% unique reads were successfully aligned to the chicken reference genome, respectively (Table 1). Based on alignment results, 1,162 new genes were aligned to NR, Swiss-Prot, GO, COG and KEGG database, of which 902 new genes were successfully annotated (Table 2; Supplementary Table 4).

**Table 1:** Comparison results between cleaned data and reference genome.

**Samples**	**Total reads**	**Mapped reads**	**Mapped ratio (%)**	**Uniq mapped reads**	**Uniq mapped ratio (%)**
C	51,214,382	40,735,159	79.54	39,604,316	77.33
M	49,751,392	39,683,786	79.76	38,618,837	77.62
H	38,010,610	30,390,881	79.95	29,655,867	78.02

**Table 2:** Statistics of new annotation gene number.

**Annotated databases**	**New gene number**	**300 ≤ length <1,000**	**Length ≥ 1,000**
COG	175	90	66
GO	453	236	165
KEGG	278	159	76
Swiss-Prot	498	254	197
nr	899	405	418
All	902	406	420

### DEGs Analysis

**Figure 1:**
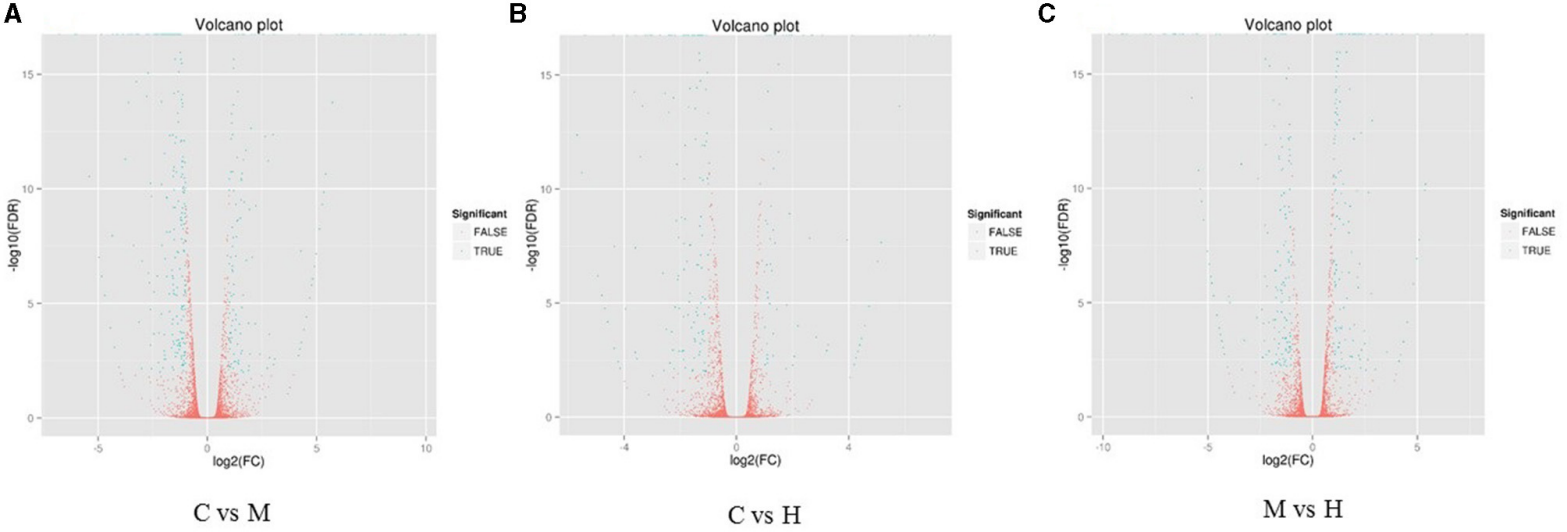
Heat maps of DEGs in the comparison between C and M **(A)**, C and H **(B)**, and M and H **(C)**. X-axis shows log_2_ (FC) of DEGs between each two samples. Y-axis indicates the –log_10_ (FDR) of gene expression variations. Each point in the volcanic map represents a gene. The green dot represents the DEGs, and the red dot represents the non-DEGs.

**Figure 2:**
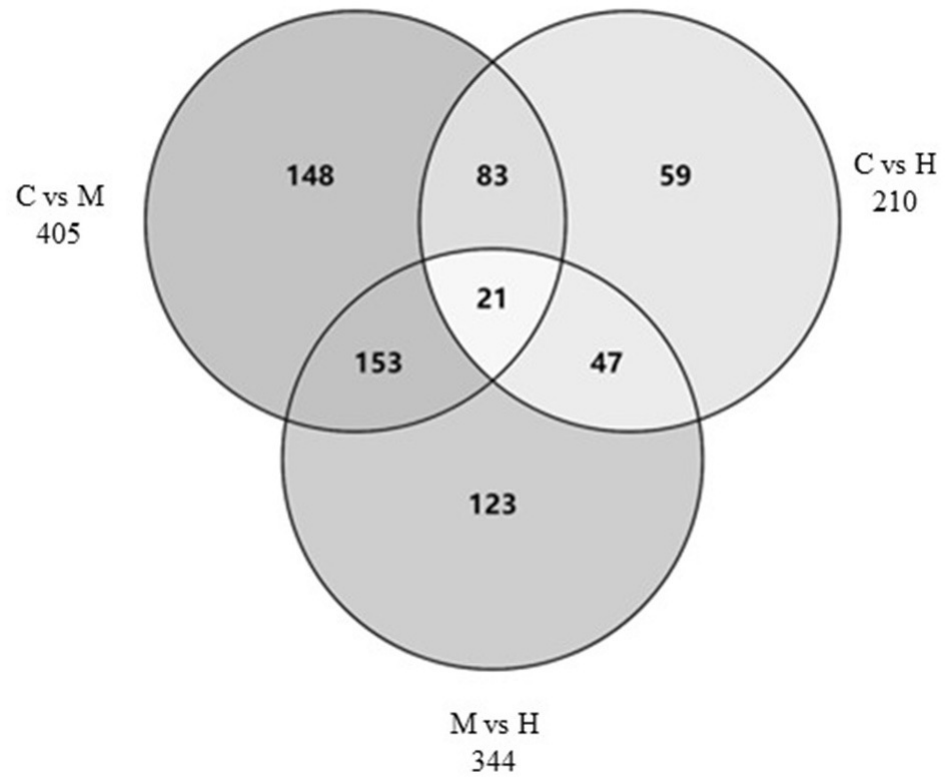
Venn diagram of DEGs among three groups.

The DEGs between different groups, i.e., C vs. M, C vs. H, and M vs. H, were screened according to a fold change of ≥2 and an FDR (False Discovery Rate) <0.01. FPKM (fragments per kilobase of transcript per million fragments mapped) was used to measure the level of transcripts or gene expression. A total of 959 DEGs were detected among three groups (Table 3; Figures 1, 2). Compared with the C group, 157 upregulated genes and 248 downregulated genes were identified in the M group. The H group had 68 upregulated genes and 142 downregulated genes compared to the C group. There were 165 upregulated genes and 179 downregulated genes between M and H groups. Of these DEGs, 903 DEGs were successfully annotated in the public databases, and the number of DEGs annotated in each functional database is shown in Table 4. The top 5 up- and downregulated DEGs between different groups are listed in Supplementary Table 5.

**Table 3:** Statistics of the number of DEGs.

**DEG Set**	**All DEG**	**Upregulated**	**Downregulated**
C vs. M	405	157	248
C vs. H	210	68	142
M vs. H	344	165	179

**Table 4:** Statistics of the number of DEGs annotated.

**DEG Set**	**Annotated**	**COG**	**GO**	**KEGG**	**Swiss-Prot**	**nr**
C vs. M	384	152	345	192	356	384
C vs. H	195	58	161	86	167	195
M vs. H	324	118	294	156	297	324

### GO Enrichment Analysis and COG Classifications

**Figure 3:**
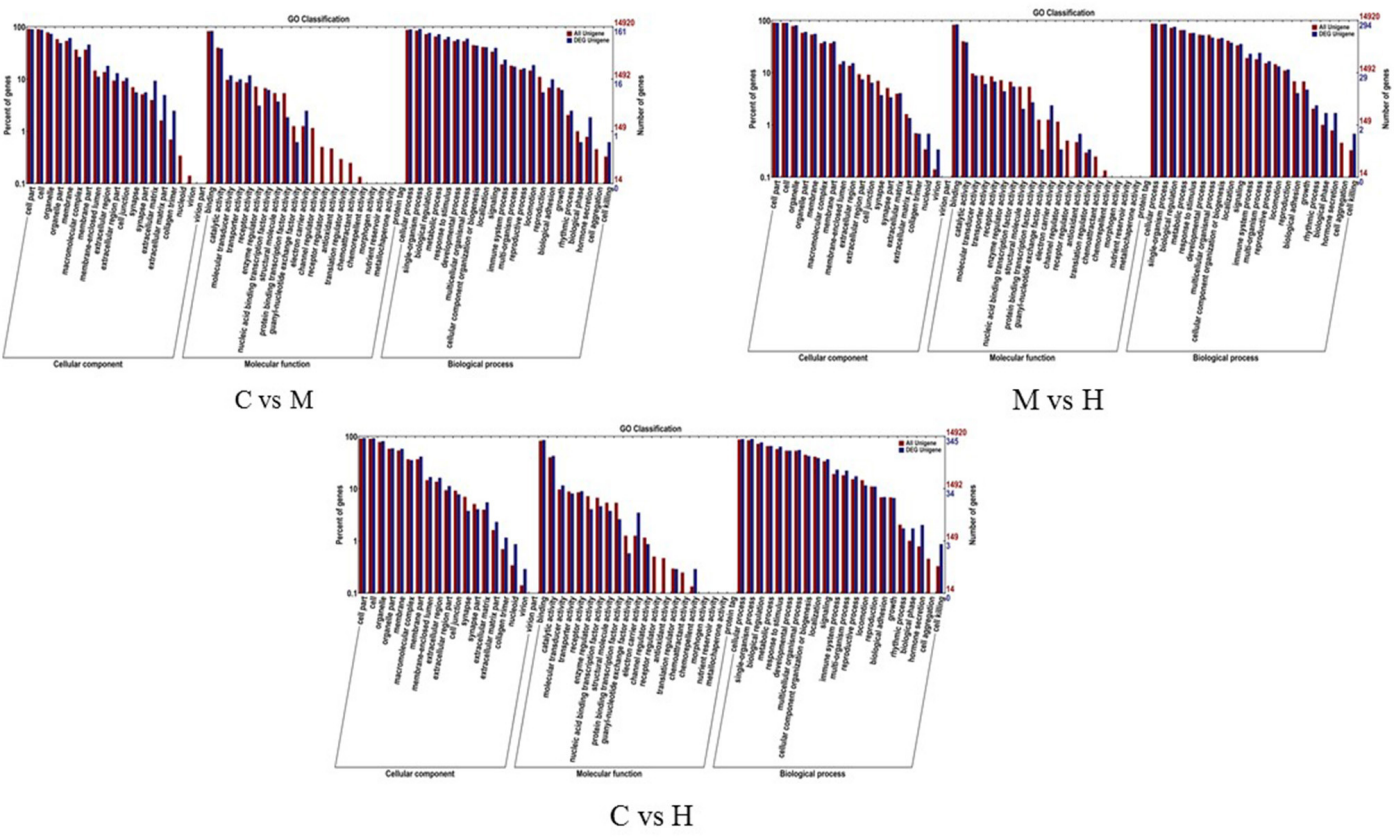
GO enrichment analysis of the DEGs. X-axis represents the sub-categories, and the left of Y-axis represents the percentage of unigenes and the right of Y-axis represents the number of unigenes in each sub-category.

To understand the role of these DEGs, GO enrichment analyses were performed to classify the putative functions of DEGs in comparison to libraries from the different groups. The results indicated that these GO terms were classified into three categories including molecular function (MF), cellular component (CC) and biological process (BP) (Table 5; Figure 3). For C vs. M groups, 405 DEGs were enriched into 4,939 GO terms. There were 2,306 enriched GO terms of 210 DEGs between C and H groups; 4,061 enriched GO terms were from 344 DEGs between M and H groups. Most genes of DEGs were related to cell part in CC, binding in MF, and single-organism process in BP (Figure 3).

**Table 5:** Summary of GO term distribution.

**Samples**	**GO term in total**	**CC**	**MF**	**BP**
C vs. M	4,939	1,843	613	2,483
C vs. H	2,306	832	280	1,194
M vs. H	4,061	1,527	481	2,053

The COG database is based on the phylogenetic relationship of bacteria, algae and eukaryotes and can be used to directly classify gene products. Based on sequence homology, 152 unigenes were subdivided into 22 functional classes (Supplementary Figure 1) in the COG database between C and M groups. Comparing C and H groups, 58 unigenes were classified into 18 functional classes (Supplementary Figure 1). Furthermore, 21 functional classes were from 118 unigenes between M and H groups (Supplementary Figure 1). Overall, “general function prediction only” represented the largest functional classes followed by “replication, recombination and repair” among three groups.

### KEGG Pathway Analysis of DEGs

**Figure 4:**
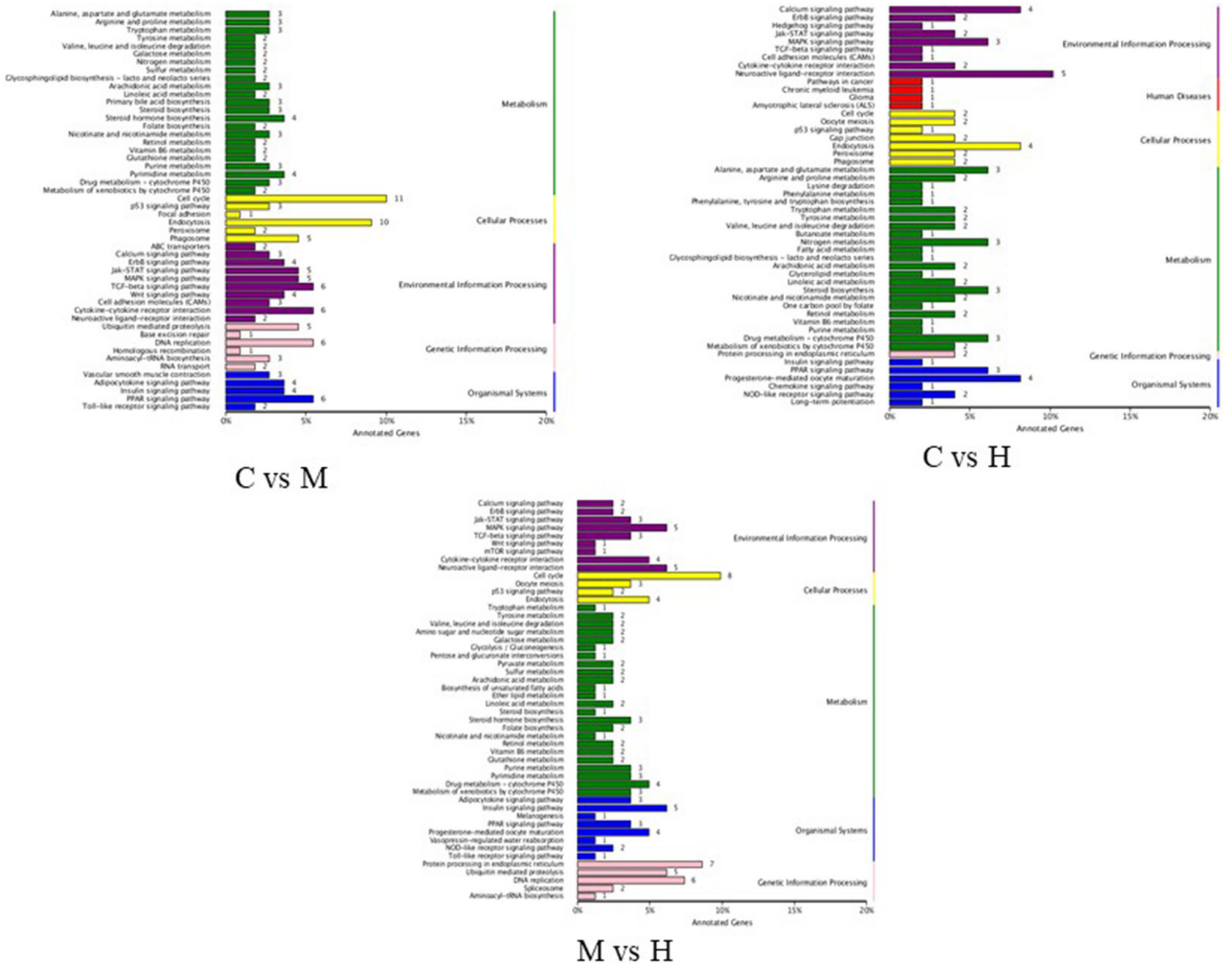
KEGG functional classification of DEGs.

KEGG pathway analysis was performed to investigate the functions of DEGs in response to folic acid. KEGG pathway annotations were obtained as shown in Figure 4. For C vs. M groups, 110 unigenes were assigned to 96 KEGG pathway, and the highest unique sequences were “cell cycle (ko04110)” followed by “endocytosis (ko04144).” For C vs. H groups, 64 KEGG pathways were found from 49 unigenes, mainly related to “neuroactive ligand-receptor interaction (ko04080).” Also, 81 unigenes were assigned to 85 KEGG pathway between M and H groups, and the largest enriched group were also “cell cycle (ko04110).” However, only some pathways were significantly enriched with *P*-values <0.05 (Supplementary Table 6). Notably, two significant pathways involved in “DNA replication” and “folate biosynthesis” were enriched in C vs. M and M vs. H groups, suggesting these may be related to folic acid metabolism in broilers. In addition, “steroid biosynthesis” and “drug metabolism - cytochrome P450” pathways were also enriched among three groups.

### SNP Analysis

According to the number of alleles (i.e., the number of different bases supported by sequencing reads) at the SNP site, the SNP locus was divided into homozygous SNP locus (only one allele) and heterozygous SNP locus (two or more alleles). The proportion of heterozygous SNP in different species was different. The number of SNP sites, proportions of conversion type, transversion type and heterozygous SNP site were screened out from each sample by SAMtools software. The SNP numbers of C, M, and H groups were 145,450, 146,131, and 123,004 (Table 6), respectively, and about 76% of the SNP sites were transition. In addition, SNP site density of each gene was counted and the density distribution was shown in Supplementary Figure 2.

**Table 6:** SNP sites.

**Samples**	**SNP number**	**Genic SNP**	**Intergenic SNP**	**Transition (%)**	**Transversion (%)**	**Heterozygosity (%)**
C	145,450	101,498	43,952	76.30	23.70	71.85
M	146,131	102,315	43,816	76.53	23.47	73.49
H	123,004	86,551	36,453	76.96	23.04	73.83

### New Alternative Splicing Event Prediction

There are many kinds of splicing methods of pre-mRNA produced by gene transcription. Different exons of genes are selected to produce different mature mRNA, which can be translated into different proteins to form the diversity of biological properties. New alternative splicing events were predicted using Cufflinks2 (24) and results were shown in Table 7. Total new predicted alternative splicing events in C, M, and H groups were 9,521, 9,328, and 8,929, respectively.

**Table 7:** The numbers of new predicted alternative splicing events.

**Samples**	**Skipped exon**	**Intron retention**	**Alt5 splice**	**Alt3 splice**	**Alt last exon**	**Alt first exon**
C	3,816	1,538	592	1,060	500	2,015
M	3,760	1,457	608	1,080	456	1,967
H	3,615	1,373	562	1,052	455	1,872

*Alt5 splice, alternative 5′ splice site; alt3 splice, alternative 3′ splice site; alt last exon, alternative last exon; Alt first exon, alternative first exon*.

### PPI Analysis

**Figure 5:**
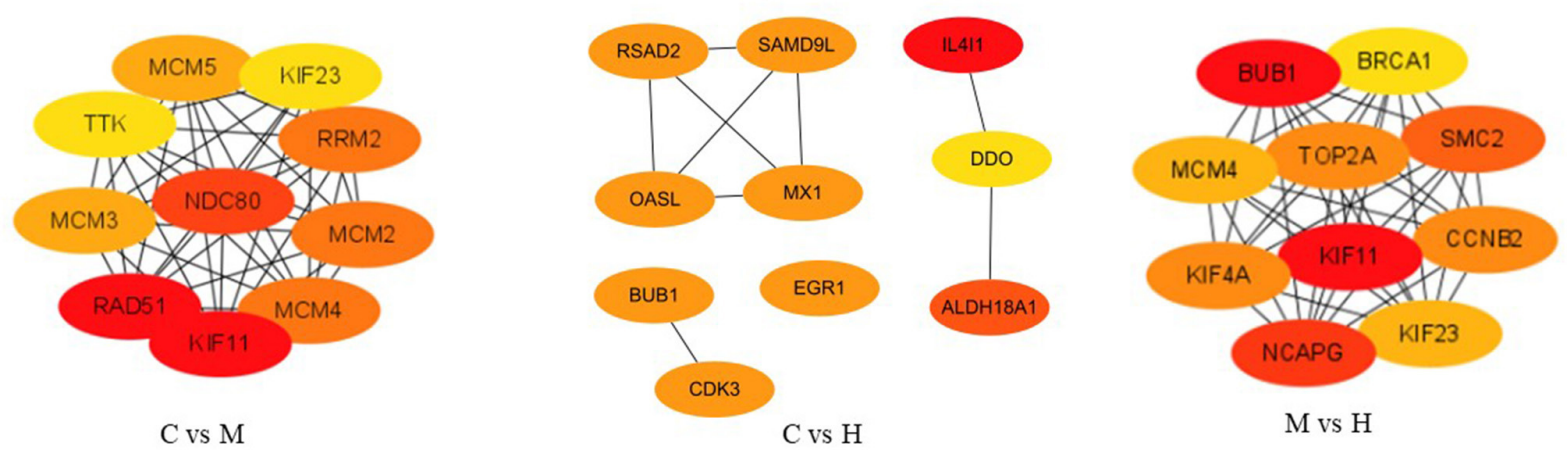
The top 10 ranked hub genes obtained from CytoHubba analysis based on degree method. The change in color from red to yellow represents a change in degree score from high to low.

After removing new genes from DEGs, 346, 161, and 292 DEGs for C vs. M, C vs. H, and M vs. H, respectively, remained for the PPI network analysis by STRING online database. A total of 198 nodes and 523 edges with a 5.28 average node degree, a 0.441 clustering coefficient, 178 expected number of edges, and a <1.e-16 PPI enrichment *p*-value were found for C vs. M groups (Supplementary Figure 3). The PPI of results between C and H groups showed 72 nodes, 30 edges with a 0.833 average node degree, a 0.329 clustering coefficient, 17 expected number of edges, and a 0.00365 PPI enrichment *p*-value (Supplementary Figure 3). The network generated for M vs. H groups was composed of 168 nodes, 630 edges with a 7.5 average node degree, a 0.544 clustering coefficient, 181 expected number of edges, and a <1.0e-16 PPI enrichment *p*-value (Supplementary Figure 3). Subsequently, the top 10 hub genes were evaluated among three groups using CytoHubba (Figure 5). The results suggest that they play important roles in folate metabolism and hepatic lipogenesis.

### Confirmation of DEGs by qPCR

**Figure 6:**
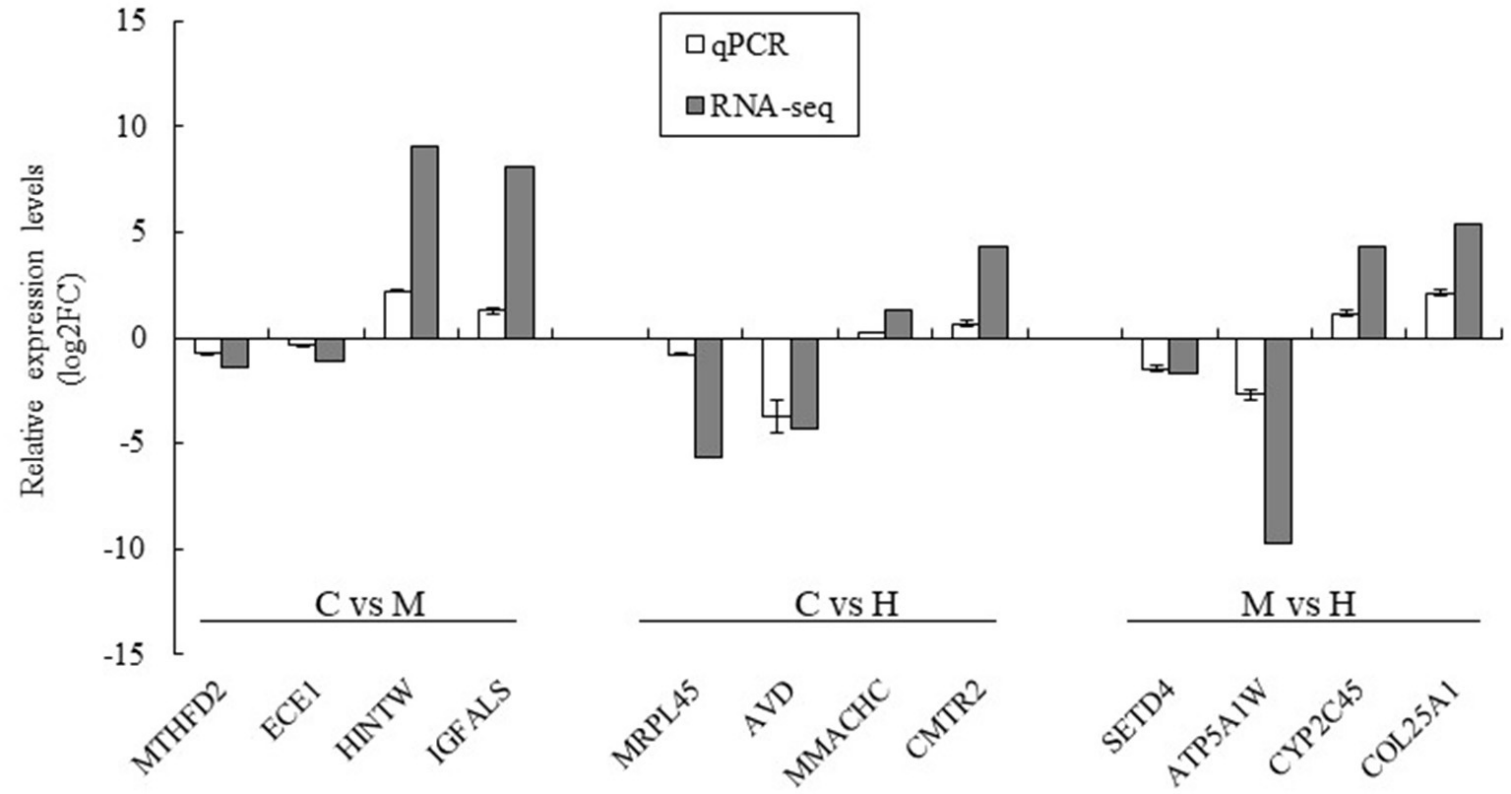
Validation of the RNA-seq results using qPCR analysis. The data was based on the fold of change of qPCR and log_2_FC of RNA-seq. MTHFD2 and ECE1 were downregulated genes and HINTW and IGFALS were upregulated genes in C vs. M groups; MRPL45 and AVD were downregulated genes and MMACHC and CMRT2 were upregulated genes in C vs. H groups; SETD4 and ATP5A1W were downregulated genes and CYP2C45 and COL25A1 were upregulated genes in M vs. H groups.

Twelve DEGs were selected to validate the results of the RNA-seq by qPCR using the same premixed RNA templates as those used in the RNA-seq analysis. A total of 12 genes related to key enzymes involved in folate metabolism were selected (Figure 6). These genes included two upregulated genes and two downregulated genes in the C vs. M, C vs. H, and M vs. H groups, respectively. The results indicated that the qPCR relative expression levels and RNA-seq results have the same tendency among all DEGs (Figure 6).

### mRNA Abundance of *LPL, PPARγ*, and *FAS* Genes

**Figure 7:**
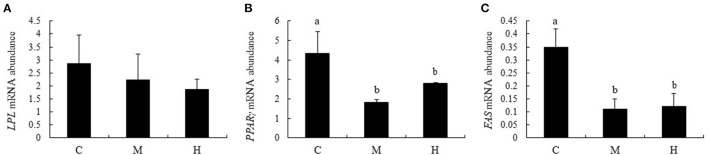
mRNA relative abundance of LPL, PPARγ, and FAS in abdominal fat of broilers. **(A–C)** represent the mRNA abundance of LPL, PPARγ and FAS genes, respectively. The mRNA relative abundance was normalized to those of β-actin gene. Values are means ± SE. Superscripts (a and b) denote significant differences (*P* < 0.05).

Effects of folic acid on expression levels of *LPL, PPAR*γ, and *FAS* genes in abdominal fat were examined using qPCR. The results showed that mRNA abundance of *PPAR*γ and *FAS* in M and H groups were reduced compared with the C group (*P* < 0.05), while *LPL* expression levels in abdominal fat were not affected by dietary FA supplementation (*P* > 0.05; Figure 7).

### Transmission Electron Microscopy

**Figure 8:**
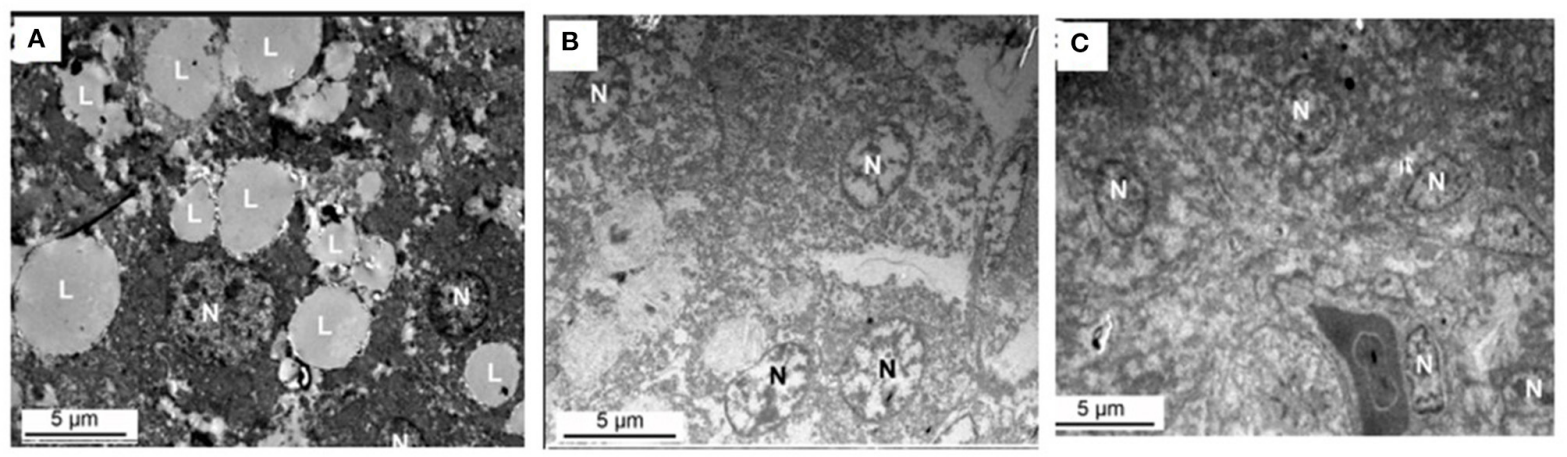
Ultramicroscopy of livers from chicken fed with or without addition of folic acid. **(A)** C group, there are a lot of large lipid droplets in the liver cell. **(B)** M group, there are few lipid droplets in the liver cell. **(C)** H group, there are few lipid droplets in the liver cell. L, lipid droplet; N, nucleus.

Figure 8 shows the effect of supplemental folate on ultramicroscopy of livers in broilers. The results indicated lipid droplets are much more in livers from chickens fed without the addition of folic acid than in livers from chickens fed with the addition of folic acid.

### Contents of LPL, PPARγ, and FAS in Abdominal Fat of Broilers

Contents of LPL, PPARγ, and FAS in abdominal fat are given in Table 8. On the whole, Contents of LPL, PPARγ, and FAS in abdominal fat were decreased with the folic acid supplmented diets (*P* < 0.01; Table 8). Moreover, the contents of LPL, PPARγ, and FAS in group M were lower than that of group H, and, the contents of PPARγ reached a significant level between group M and group H (*P* < 0.01; Table 8).

**Table 8:** Effects of folic acid supplementation in basal diets on LPL, PPARγ, and FAS contents in abdominal fat of broilers (*n* = 6).

**Items**	**C group**	**M group**	**H group**	***P-*value**
LPL (IU/L)	395.36 ± 8.21^A^	331.41 ± 5.48^B^	357.09 ± 5.50^B^	<0.0001
PPARγ (ng/L)	542.36 ± 10.45^A^	401.33 ± 9.31^B^	462.05 ± 11.62^C^	<0.0001
FAS (nmol/L)	23.11 ± 1.23^A^	14.75 ± 0.96^B^	16.69 ± 1.02^B^	<0.0001

*In the same row, values with different capital letter superscripts mean significant difference (P < 0.01)*.

## Discussion

The liver is considered as the primary site of lipogenesis in birds (3), and folic acid metabolism in liver is a very complex process (8). In present study, we have identified 959 DEGs from three groups and 903 DEGs were annotated in the public databases. Among these DEGs, 390 DEGs were upregulated gene, and 569 DEGs were downregulated genes. Among these DEGs, 12 key genes were selected to confirm the accuracy of RNA-seq by qPCR, and the direction of expression of these genes assayed by qPCTR was consistent with RNA-seq, suggesting that RNA-seq is a reliable method for genome-wide analysis of expression of profiles. In addition, some fat metabolism related genes (*LPL, PPAR*γ, *FAS*) expression levels and their contents in abdominal fat, and size differences in liver lipid droplets were also obsered among three groups, respectively. This study provides some evidence to a better understanding of the molecular mechanism of folic acid regulating fat metabolism.

Based on functional and GO enrichment analyses of the DEGs among different groups, methylenetetrahydro folate dehydrogenase 2 (*MTHFD2*) and nidogen-1 (*NID1*) genes, which were associated with folic acid metabolism, were downregulated by folic acid supplementation. More importantly, MTHFD2 is a bifunctional mitochondrial one-carbon folate metabolic enzyme with methylene dehydrogenase, catalyzing the NAD^+^ dependent 5,10-methylene-THF (tetrahydrofolate) dehydrogenase and 5,10-methenyl-THF cyclohydrolase reactions within the mitochondria (29–31). MTHFD2 was found in the nucleus, and co-localize with DNA replication sites (32). It was reported that *MTHFD2* is expressed in the developing embryo, but is absent in most healthy adult tissues (30), and overexpression of *MTHFD2* alone was sufficient to promote tumor cell proliferation (32). Studies have shown that elevated *MTHFD2* expression is associated with poor prognosis in both hematological and solid malignancy (31). *NID1* is a member of the nidogen family of basal membrane glycoproteins (33), and it probably has a role in cell-extracellular matrix interactions (34). Recently, a study showed that suppression of *NID1* expression reduces proliferation and migration/invasion in claudin-low murine mammary tumor cells (35). Our results indicated that folic acid inhibited the expression of *MTHFD2* and *NID1*, speculating that folic acid may inhibit the expression of *MTHFD2* and *NID1*, and it may be used in cancer treatment in the future.

Moreover, functional analyses found that some DEGs associated with methyltransferase activity such as SETMAR and CYP51A1, were also downregulated by folic acid. SETMAR is a protein methylase with a sequence-specific DNA binding domain and is expressed in most tissues and cells, and it plays a role in DNA recombination, repair and chromosome decatenation (36). Most importantly, expression of *SETMAR* increased in leukemias, breast cancer and glioblastoma (37–39). CYP51A1, or lanosterol 14α-demethylase, is the essential enzyme that catalyzes an early stage of cholesterol biosynthesis and is present in all biological kingdoms (40, 41). A study has shown that innate immune transcriptional downregulation of *CYP51A1* induces lanosterol accumulation in macrophages, promoting antimicrobial activity and favoring anti-inflammatory response in macrophages (42).

In addition to the above genes downregulated by folic acid, some DEGs were upregulated by adding folic acid to diets, such as *UHRF1, FTSJD1, RAD51A, MCM3, PHF19, RCA1*, and *MYBL1*. These DEGs were involved in DNA (mRNA) methylation, macromolecule methylation, methyl-CpG binding, methylated histone binding, histone H3-K4 methylation, methylation-dependent chromatin silencing, mRNA (nucleoside-2'-O-)-methyltransferase activity and so on. Notably, *UHRF1*, an epigenetic modulator, is genetically linked to DNA methylation maintenance and histone modifications (43, 44). Another gene, *FTSJD1*, which is distributed in the nucleus and cytoplasm of humans, catalyzes ribose methylation of recombinant RNA and plays an important role in the regulation of gene expression, including enhancing RNA stability, splicing, nucleoplasmic transport, and translation initiation, as well as promoting interactions between the nucleus and cytoplasmic cap-binding proteins (45). As a methyl donor, folic acid can directly participate in the synthesis and maintenance of RNA, DNA, and protein as well as DNA methylation and epigenetic modification (46, 47). Thus, folic acid may affect the expression of *UHRF1* and *FTSJD1* by regulating the methylation level of *UHRF1* and *FTSJD1*.

KEGG pathway analysis identified 35 significantly enriched pathways of DEGs among groups. Interestingly, these enriched pathways are mainly associated with folate biosynthesis, *PPAR* signaling pathway, cell cycle, DNA replication, steroid biosynthesis, vitamin B6 metabolism, TGF-beta signaling pathway, etc (Supplementary Table 6). The folate biosynthesis (ko00790) is an attractive pathway for the development of new therapies against some human diseases, including cancer and rheumatoid arthritis, which involves several steps and many enzymes, such as GTPCHI, DHNP, DHPS, and DHFR (48). The *PPAR* signaling pathway, a critical pathway in lipid metabolism, regulates the expression of many genes involved in glucose and lipid homeostasis, inflammation, proliferation, and differentiation (49–51). Studies have shown that it may be an important predictor of breast cancer and regulated placental lipid metabolism (50, 52, 53). In chickens, some studies have also indicated that enrichment of the *PPAR* signaling pathway helps in adaptation during adverse conditions such as heat stress, and is required for energy metabolism and regulating the oxidative stress-induced inflammatory response (2, 51, 54). Similarly, it was reported that paternal dietary folate supplementation leads to the transgenerational inheritance of acquired glucose and lipid metabolic changes in broilers owing to the altered hepatic gene expression and changes in the *PPAR* signaling pathway, the FoxO signaling pathway, etc. (19). Thus, our results presented suggest that these enriched pathways of DEGs may play an important role in regulating gene expression, DNA methylation, folate, and lipid metabolism.

SNPs mainly refers to the DNA sequence polymorphism caused by single nucleotide variation at the genomic level. At present, the 2.8 million SNPs were identified using whole-genome sequencing of multiple chicken breeds, which at least 90% of the variant sites are true SNPs, and at least 70% are common SNPs that segregate in many domestic breeds (55). It was reported that selection could afected the frequency of SNPs in positional candidate genes leading to changes in lipogenesis and lipid accumulation in chickens (4). In present study, a total of 414,585 SNPs were screened (Table 6). Most of these SNPs are transition and heterozygosity. Whether these SNPs in positional candidate genes are related to liver fat and folic acid metabolism needs further experimental verification.

Until now, some studies are available regarding the effects of folic acid reducing lipid accumulation in 3T3-L1 adipocytes (56), chicken hepatocytes (17, 57), and broilers (18). These results suggest that folic acid nutrients have the potential to regulate lipid metabolism. In present study, broiler chicks were fed with different levels of folic acid to study the effects of folic acid on the morphology of liver fat cells and the gene expression of liver fat metabolism. Our results showed that expression levels of *PPAR*γ and *FAS* in abdominal fat and the size of lipid droplets in liver was decreased by the addition of folic acid in food. On the other hand, our previous study has found that the folate-deficient food of broiler breeders remarkably increased the size of the lipid droplets and the expression of *LPL* and *IGF2* genes in liver of offspring (20). Conversely, previous studies indicated that folic acid increased the expression of *LPL* and *IGF2* genes and decreased the methylation level of *IGF2* gene in liver of broilers (18, 58). The differences in the *LPL* and *IGF2* expression levels may be due to the different physiological stages of the chickens. Recently, Zhang et al. found that supplemented folic acid in palm fat powder treated diets could reduce fat accumulation in the liver of Holstein dairy bulls by down-regulating hepatic mRNA expression of *FAS* (59). This is consistent with our results. As described above, folic acid can be decrease hepatic lipogenesis by regulating the expression of some genes related to fat metabolism.

## Conclusions

In summary, this study reported the gene expression responses to folic acid supplement in broilers using RNA-seq. A total of 959 DEGs were detected among three groups. These genes belonged to pathways and gene networks related to folic acid metabolism, hepatic lipogenesis, DNA methylation, and methyltransferase activity. And present study also found that several metabolic pathways such as folate biosynthesis, *PPAR* signaling pathway, DNA replication, etc, are modulated by folic acid supplementation. These findings suggest that folic acid may reduce fat accumulation by regulating gene expression, and provide scientific basis for the future research on the application of folic acid in poultry.

## Data Availability Statement

The datasets presented in this study can be found in online repositories. The names of the repository/repositories and accession number(s) can be found below: https://www.ncbi.nlm.nih.gov/bioproject/PRJNA719583, PRJNA719583.

## Ethics Statement

The animal study was reviewed and approved by the experiment was conducted at Linyi University in China, and all animal experimental protocols were approved by the Animal Care and Use Committee. Written informed consent was obtained from the owners for the participation of their animals in this study.

## Author Contributions

YZ and JX performed the experiments and analyzed the data. JX and XL designed the experiments. NZ assayed gene expression. LL performed transmission electron microscopy. YW analyzed the data. JX wrote the manuscript. All the authors read and approved the final version of the manuscript.

## Funding

This research was funded by the Nature Science Foundation of Shandong Province of China (Grant Number ZR2017LC018), the National Natural Science Foundation of China (Grant Number 31372333), and Linyi Key Research and Development Project (Grant Number 2020ZX028).

## Conflict of Interest

The authors declare that the research was conducted in the absence of any commercial or financial relationships that could be construed as a potential conflict of interest.

## Publisher's Note

All claims expressed in this article are solely those of the authors and do not necessarily represent those of their affiliated organizations, or those of the publisher, the editors and the reviewers. Any product that may be evaluated in this article, or claim that may be made by its manufacturer, is not guaranteed or endorsed by the publisher.

## Supplementary Material

Supplementary Figure 1 Classification of COG annotation of DEGs. X-axis represents function class, Y-axis represents the number of genes.

Supplementary Figure 2 SNP density distribution. The x-axis represents the numbers of SNPs distributed in average per kb, and the y-axis represents the frequency of genes.

Supplementary Figure 3 The size of a node in the interaction network is proportional to the degree of this node, that is, the more edges connected to this node, the greater its degree, and the larger the node. The color of a node is related to the clustering coefficient of the node, and the color gradient from green to red corresponds to the clustering coefficient from low to high. The clustering coefficient indicates the connectivity between the adjacent points of this node. The higher value of the clustering coefficient, the better the connectivity between the adjacent points of this node. The width of an edge indicates the strength of the interaction between the two nodes connected by this edge. The stronger the interaction, the wider the edge. No combination means no interaction.

Supplementary Table 1 Content and nutrition level of diet for broiler (air-dry basis, %).

Supplementary Table 2 Primers used for qPCR.

Supplementary Table 3 Sample sequencing data assessment statistics.

Supplementary Table 4 New genes were successfully annotated.

Supplementary Table 5 Top 5 up and down regulated DEGs according to log_2_FC value among groups.

Supplementary Table 6 Significantly enriched pathways of DEGs among groups.
