# Supplementary figures and images for: Transcriptome Analysis of Effects of Folic Acid Supplement on Gene Expression in Liver of Broiler Chickens
Source: Front Vet Sci. 2021 Sep 16;8:686609. doi: 10.3389/fvets.2021.686609 (PMC8481781; doi:10.3389/fvets.2021.686609)

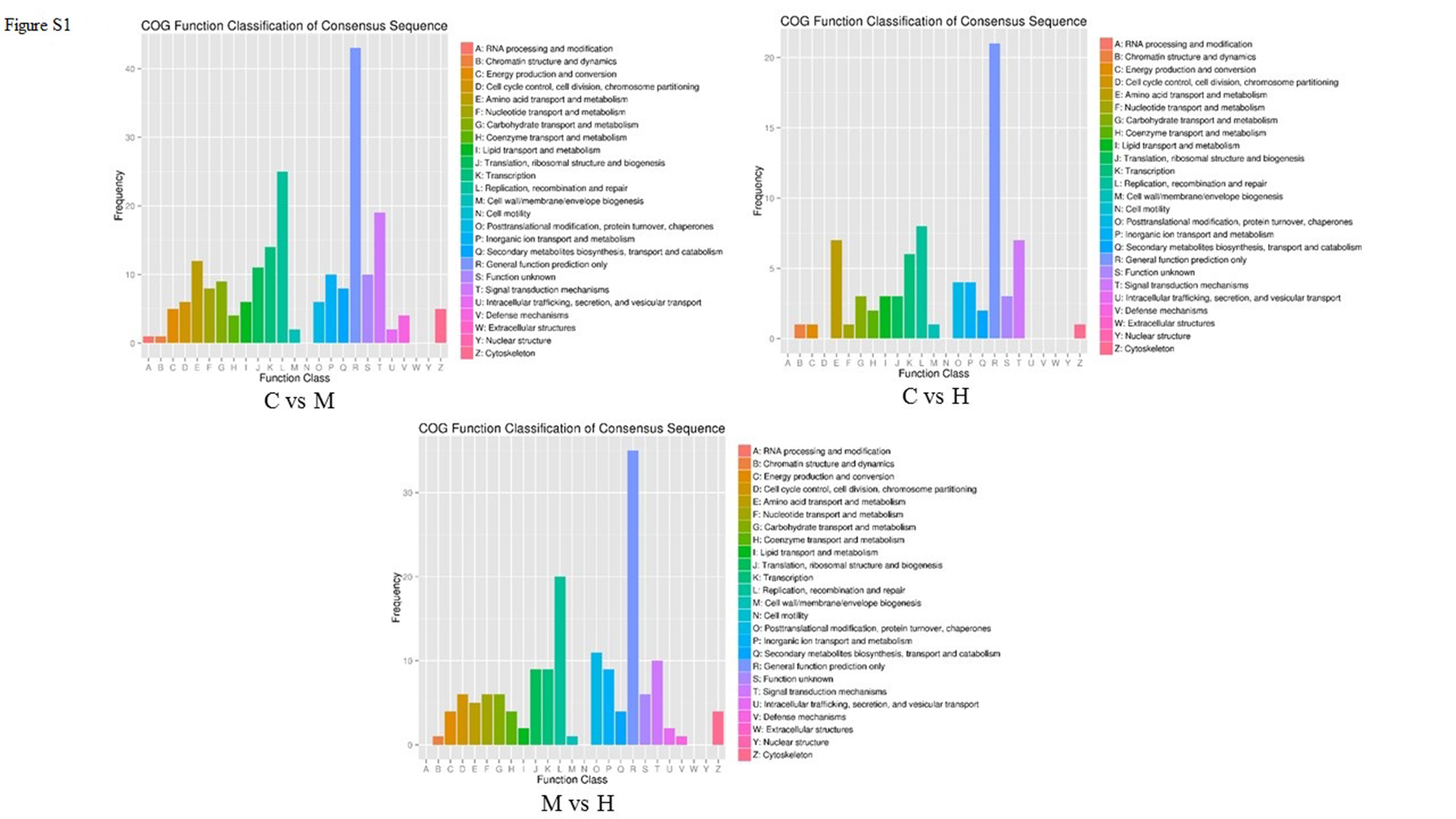

Supplement: Supplementary Figure 1 — Classification of COG annotation of DEGs. X-axis represents function class, Y-axis represents the number of genes. [file Image_1.jpg]

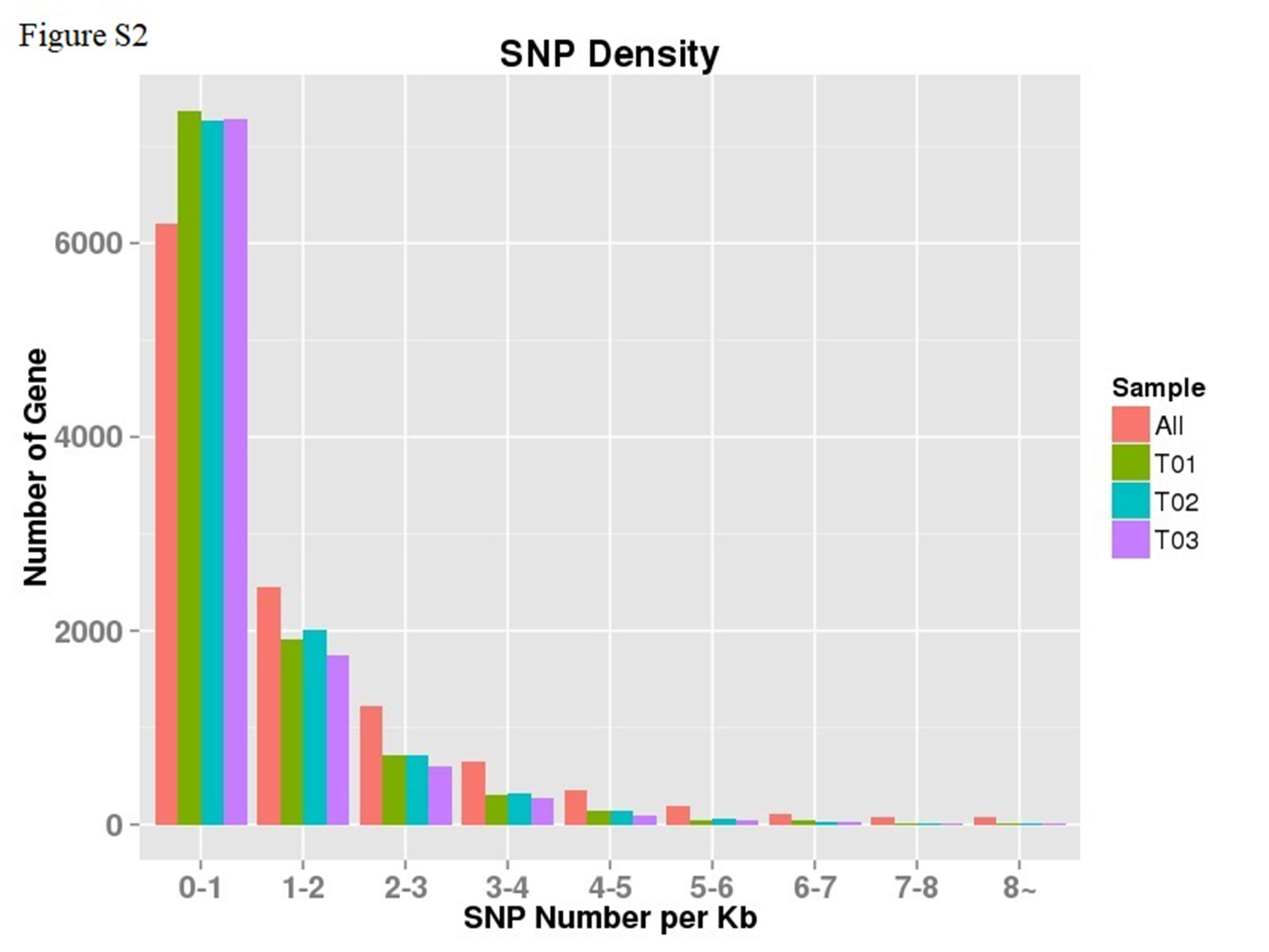

Supplement: Supplementary Figure 2 — SNP density distribution. The x-axis represents the numbers of SNPs distributed in average per kb, and the y-axis represents the frequency of genes. [file Image_2.jpg]

C vs M

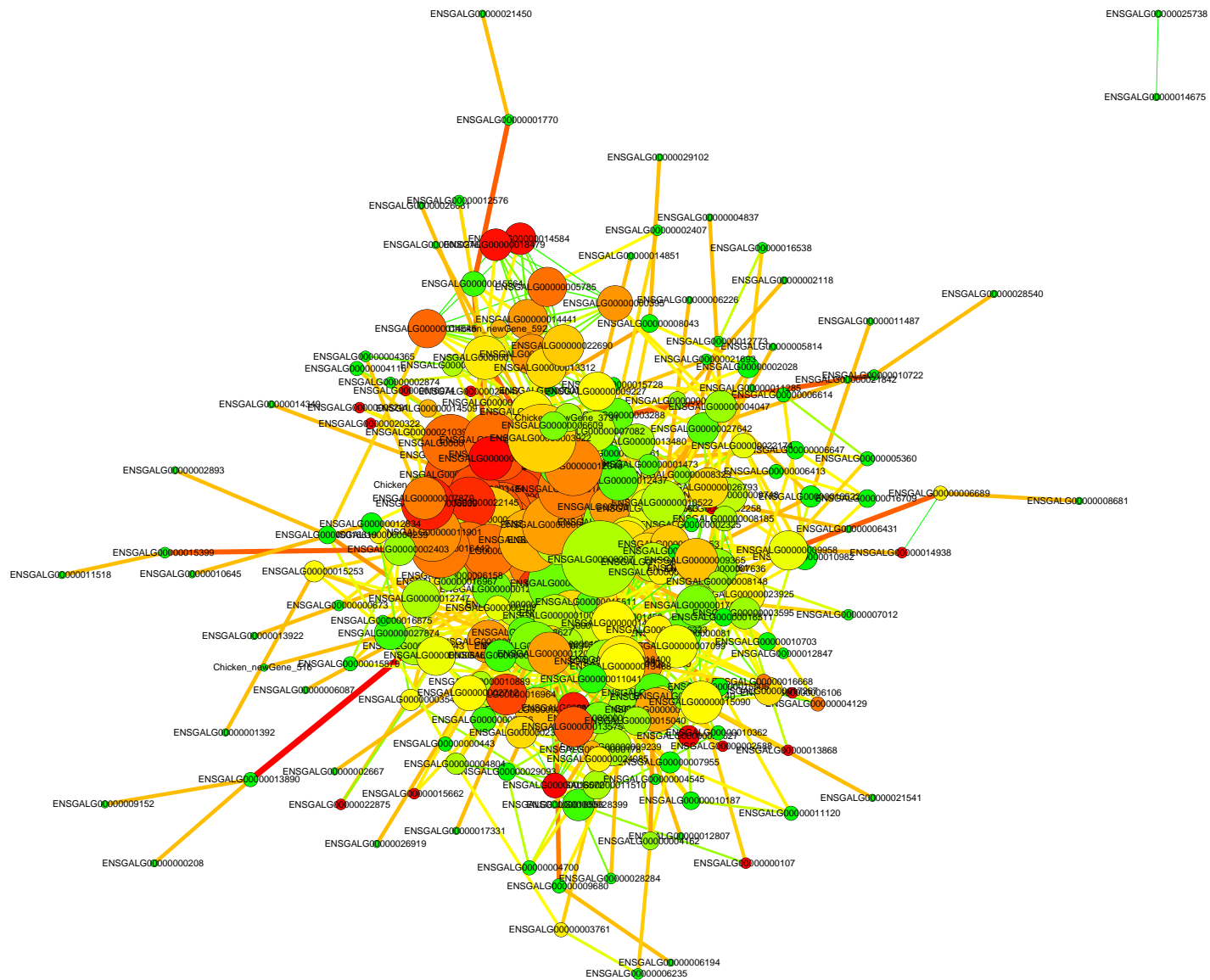

C vs H

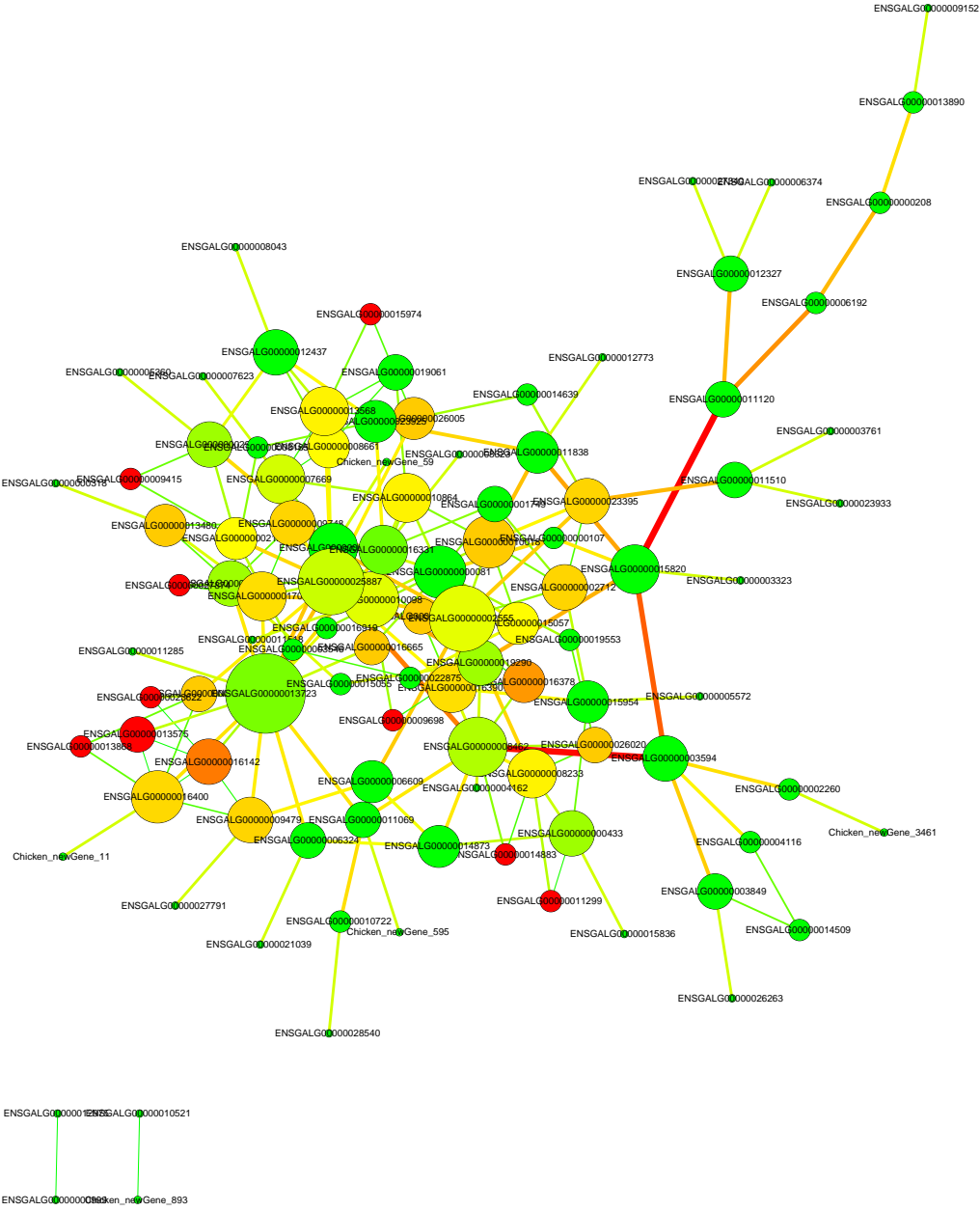

M vs H

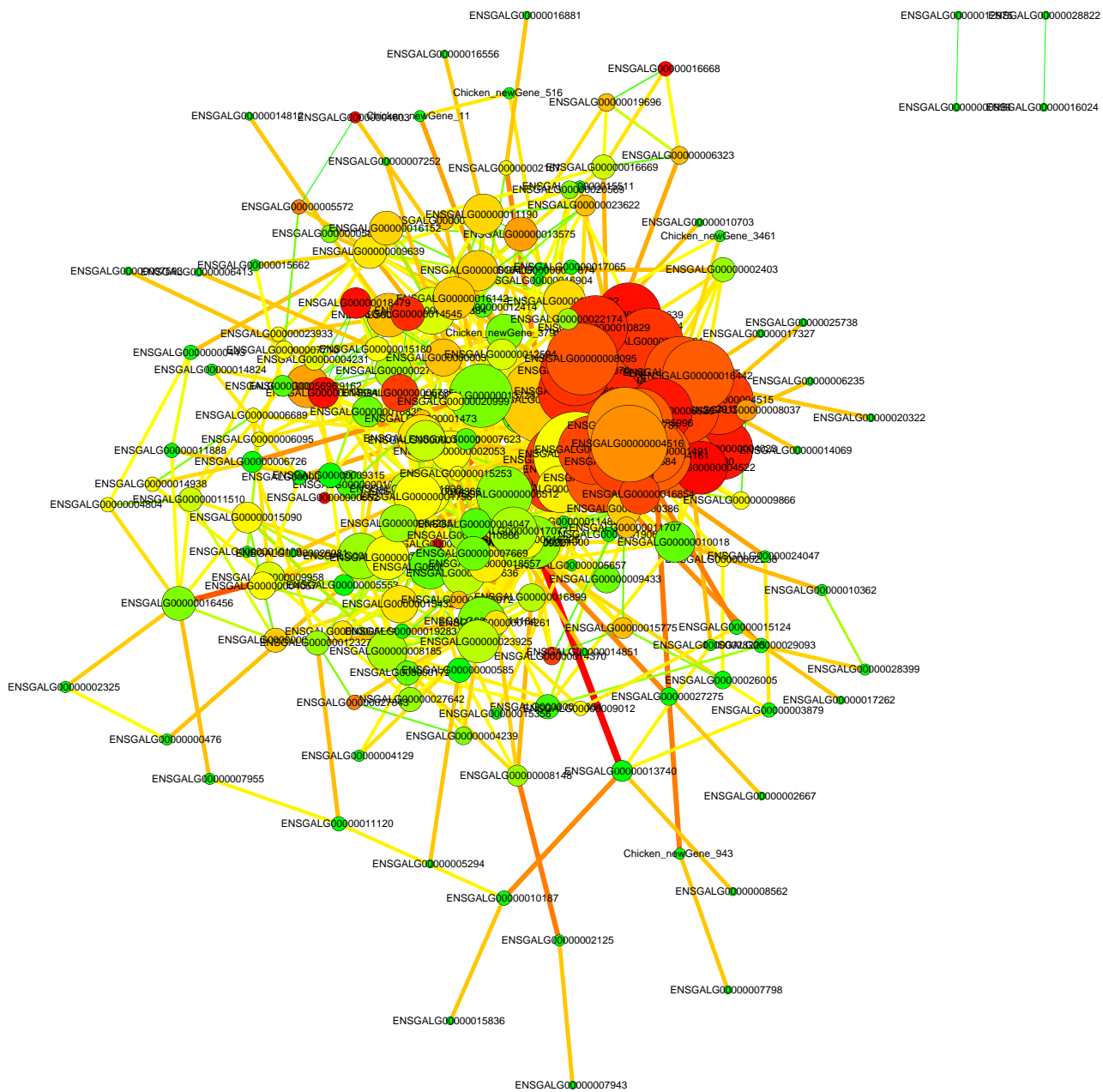

Supplement: Supplementary Figure 3 — The size of a node in the interaction network is proportional to the degree of this node, that is, the more edges connected to this node, the greater its degree, and the larger the node. The color of a node is related to the clustering coefficient of the node, and the color gradient from green to red corresponds to the clustering coefficient from low to high. The clustering coefficient indicates the connectivity between the adjacent points of this node. The higher value of the clustering coefficient, the better the connectivity between the adjacent points of this node. The width of an edge indicates the strength of the interaction between the two nodes connected by this edge. The stronger the interaction, the wider the edge. No combination means no interaction. [file Image_3.pdf]
