# Supplementary material for: Transcriptome Analysis of Effects of Folic Acid Supplement on Gene Expression in Liver of Broiler Chickens
Source: Front Vet Sci. 2021 Sep 16;8:686609. doi: 10.3389/fvets.2021.686609 (PMC8481781; doi:10.3389/fvets.2021.686609)
Supplement: Supplementary Table 1 — Content and nutrition level of diet for broiler (air-dry basis, %). [file Table_1.DOCX]

**Table s1** Content and nutrition level of diet for broiler (air-dry basis, %)

| **Items** | **1-21 days** | **21-42 days** |
| --- | --- | --- |
| Material Ingredient (%) | | |
| Corn | 54.50 | 57.60 |
| Soybean | 32.71 | 30.42 |
| Fish meal | 3.00 | 2.00 |
| Shell powder | 1.22 | 1.05 |
| CaHPO4·2H2O | 1.70 | 1.60 |
| Soybean oil | 3.00 | 4.00 |
| Wheat bran | 2.50 | 2.00 |
| Salt | 0.20 | 0.19 |
| DL-Methionine (98%) | 0.17 | 0.14 |
| Premix compound ^1)^ | 1.00 | 1.00 |
| Total | 100.00 | 100.00 |
| Calculated nutrient Level ^2)^ | | |
| Apparent metabolic energy (MJ/kg) | 12.52 | 12.74 |
| Crude protein (%) | 21.52 | 20.05 |
| Calcium (%) | 0.97 | 0.89 |
| Available phosphorus (%) | 0.45 | 0.40 |
| Lysine (%) | 1.21 | 1.18 |
| Methionine (%) | 0.53 | 0.47 |
| Methionine+cystine (%) | 0.91 | 0. 82 |
| Folic acid | 0.36 | 0.33 |

1. *Premix provided per kilogram of diet: Mn, 55 mg; Zn, 55 mg; Fe, 44 mg; Cu, 5.5 mg; I, 0.44 mg; Se, 0.099 mg; VA, 770 IU; VB1, 1.2 mg; VB2, 4.8 mg; VB6, 1.6 mg; VB12, 0.011 mg; VD3, 255 IU; VE, 15 mg; VK, 2.2 mg; pantothenic acid, 11 mg; niacin, 35.5 mg; folic acid, 0.66 mg; biotin, 0.11 mg; choline, 500 mg; xanthin, 33.1 mg; antioxidant, 120 mg.*
2. *2) Nutrient level=* *the content of a nutrient in component 1 of the diet × the proportion of the component in the diet+* *the content of a nutrient in component 2 of the diet × the proportion of the component in the diet+ ………the content of a nutrient in component n of the diet × the proportion of the component in the diet.*

**TABLE S2** Primers used for qPCR

| **Gene** | **Primer sequence (5'→3')** | **Amplicon size (bp)** | **Annealing temperature (°C)** |
| --- | --- | --- | --- |
| *MTHFD2* | GATGTGGGAATCAGCAGT | 57 | 145 |
|  | GCTCGTCAATGTGTTCTG |  |  |
| *ECE1* | TTGGAGTTTGAGACAGCG | 189 | 60 |
|  | ATACACCACCACAGGCTCT |  |  |
| *HINTW* | GGCGTTTGATGATTGTTGG | 93 | 60 |
|  | AAGGGACTGAGGGTGGGTAT |  |  |
| *IGFALS* | TGGACATCTCCCAGAACA | 177 | 57 |
|  | AGGAAGGGCACAAGAATC |  |  |
| *MRPL45* | ATGGTCCGTGGCAATAGGT | 185 | 61 |
|  | TACATCAGCCTCCCAAAGC |  |  |
| *AVD* | ACATCACAGCCGTAACAG | 141 | 58 |
|  | TGAAGACAGTGGTGGACT |  |  |
| *MMACHC* | GAGTTGCTGGAGTTGTTC | 123 | 55 |
|  | GAATCTTTCTGCTGGAGG |  |  |
| *CMTR2* | TGAACTGCTGCTTTGAGG | 107 | 58 |
|  | AATGCTCTCTCTGCCCAT |  |  |
| *SETD4* | GGCAGGAGAACTGGTTAT | 113 | 58 |
|  | AAGGAGATACAGGAGGCT |  |  |
| *ATP5A1W* | TCTCTGACTGCTTTGCCTG | 196 | 58 |
|  | GTTTCATTGCCCTGGTCT |  |  |
| *CYP2C4* | CTTTGTGGCAGAAGAGGT | 185 | 59 |
|  | TGCTTGTTGTCTCCGTTC |  |  |
| *COL25A1* | GGTTTCATTGGTCCACAG | 111 | 55 |
|  | AAGTCCAGTTTCACCTCG |  |  |
| *LPL* | TCATTGTTGTGGACTGGC | 58 | 139 |
|  | TGGACATTGTTGAGAGGGT |  |  |
| *PPARγ* | TCTCCTGGCTTCTCTCAT | 57 | 116 |
|  | TGGGCTCCATAAAGTCAC |  |  |
| *FAS* | TGAAGGAGGAAGTCAACG | 55 | 196 |
|  | GATGGTGAGGAGTCGGAT |  |  |
| *β-actin* | GTGACATCAAGGAGAAGC | 105 | 55 |
|  | CATCAGGGAGTTCATAGC |  |  |

**TABLE S3** Sample sequencing data assessment statistics

| **Samples** | **Read number** | **Base number** | **GC content** | **%≥Q30** |
| --- | --- | --- | --- | --- |
| C | 25,607,191 | 6,426,738,160 | 50.13% | 89.10% |
| M | 24,875,696 | 6,243,223,768 | 49.72% | 89.05% |
| H | 19,005,305 | 4,767,204,383 | 49.84% | 89.26% |

**Table S5** Top 5 up and down regulated DEGs according to log2FC value among groups.

| **Samples** | **Gene ID** | **FDR** | **log2FC** | **Regulated** | **nr annotation** |
| --- | --- | --- | --- | --- | --- |
| C vs M | ENSGALG00000001756 | 0 | 9.654 | up | PREDICTED: ATP synthase subunit alpha, mitochondrial isoform X2 |
|  | ENSGALG00000022685 | 0 | 9.073 | up | Wpkci |
|  | ENSGALG00000000386 | 0 | 9.008 | up | PREDICTED: transitional endoplasmic reticulum ATPase-like, partial |
|  | ENSGALG00000022679 | 0 | 8.855 | up | histidine triad missing |
|  | ENSGALG00000002325 | 0 | 8.087 | up | PREDICTED: insulin-like growth factor binding protein, acid labile subunit isoform X3 |
|  | ENSGALG00000028399 | 0 | -6.768 | down | Ig V-region-like antigen precursor |
|  | ENSGALG00000025779 | 2.85E-11 | -5.397 | down | PREDICTED: microtubule-actin cross-linking factor 1-like, partial |
|  | Chicken_newGene_3638 | 0 | -4.855 | down | PREDICTED: zonadhesin-like |
|  | ENSGALG00000011518 | 6.58E-07 | -4.829 | down | glycosyltransferase-like domain-containing protein 2 precursor |
|  | ENSGALG00000012576 | 4.52E-06 | -4.692 | down | PREDICTED: ras and EF-hand domain-containing protein isoform X1 |
| C vs H | ENSGALG00000016390 | 0 | 7.037 | up | PREDICTED: zinc fingers and homeoboxes protein 2 isoform X4 |
|  | ENSGALG00000010722 | 0 | 6.867 | up | Schwann cell-specific EGF-like repeat autocrine factor precursor |
|  | Chicken_newGene_59 | 0 | 6.467 | up | PREDICTED: GRB2-associated-binding protein 2 isoform X1 |
|  | Chicken_newGene_1500 | 0 | 6.189 | up | PREDICTED: small integral membrane protein 7, partial |
|  | ENSGALG00000011354 | 2.30E-14 | 5.799 | up | beta-crystallin A2 |
|  | Chicken_newGene_3461 | 4.19E-13 | -5.672 | down | 39S ribosomal protein L45, mitochondrial |
|  | ENSGALG00000025779 | 1.93E-11 | -5.506 | down | PREDICTED: microtubule-actin cross-linking factor 1-like, partial |
|  | ENSGALG00000019061 | 0 | -5.404 | down | PREDICTED: interstitial collagenase |
|  | ENSGALG00000011518 | 6.36E-07 | -4.937 | down | glycosyltransferase-like domain-containing protein 2 precursor |
|  | ENSGALG00000011962 | 4.62E-06 | -4.799 | down | PREDICTED: protein NDNF isoform X6 |
| M vs H | ENSGALG00000028399 | 0 | 7.362 | up | Ig V-region-like antigen precursor |
|  | ENSGALG00000027901 | 0 | 5.676 | up | thyroid hormone responsive spot 14 beta |
|  | ENSGALG00000022531 | 6.53E-11 | 5.388 | up | PREDICTED: UPF0444 transmembrane protein C12orf23 homolog |
|  | ENSGALG00000010521 | 1.21E-10 | 5.358 | up | PREDICTED: collagen alpha-1(XXV) chain isoform X2 |
|  | Chicken_newGene_58 | 1.79E-08 | 5.090 | up | hypothetical protein Anapl_17450, partial |
|  | ENSGALG00000001756 | 0 | -9.692 | down | PREDICTED: ATP synthase subunit alpha, mitochondrial isoform X2 |
|  | ENSGALG00000022685 | 0 | -9.112 | down | Wpkci |
|  | ENSGALG00000000386 | 0 | -9.047 | down | PREDICTED: transitional endoplasmic reticulum ATPase-like, partial |
|  | ENSGALG00000022679 | 0 | -8.894 | down | histidine triad missing |
|  | ENSGALG00000022674 | 0 | -7.969 | down | Wpkci |

**Table S6** Significantly enriched pathways of DEGs among groups.

| **Samples** | **KEGG pathway** | **Ko id** | **DEGs associated with the pathway (%)** | **All genes with pathway annotation (%)** | **P-value** |
| --- | --- | --- | --- | --- | --- |
| C vs M | Cell cycle | ko04110 | 11 (10.00%) | 111 (2.603%) | 0.0001 |
|  | DNA replication | ko03030 | 6 (5.455%) | 31 (0.727%) | 0.0001 |
|  | Primary bile acid biosynthesis | ko00120 | 3 (2.727%) | 13 (0.305%) | 0.0040 |
|  | PPAR signaling pathway | ko03320 | 6 (5.455%) | 60 (1.407%) | 0.0042 |
|  | Steroid hormone biosynthesis | ko00140 | 4 (3.636%) | 29 (0.680%) | 0.0061 |
|  | TGF-beta signaling pathway | ko04350 | 6 (5.455%) | 72 (1.689%) | 0.0101 |
|  | Steroid biosynthesis | ko00100 | 3 (2.727%) | 18 (0.422%) | 0.0103 |
|  | Vitamin B6 metabolism | ko00750 | 2 (1.818%) | 8 (0.188%) | 0.0167 |
|  | Nicotinate and nicotinamide metabolism | ko00760 | 3 (2.727%) | 23 (0.539%) | 0.0203 |
|  | Sulfur metabolism | ko00920 | 2 (1.818%) | 9 (0.211%) | 0.0211 |
|  | Folate biosynthesis | ko00790 | 2 (1.818%) | 10 (0.235%) | 0.0259 |
|  | Endocytosis | ko04144 | 10 (9.091%) | 200 (4.690%) | 0.0323 |
|  | Alanine, aspartate and glutamate metabolism | ko00250 | 3 (2.727%) | 30 (0.704%) | 0.0409 |
|  | Drug metabolism - cytochrome P450 | ko00982 | 3 (2.727%) | 32 (0.750%) | 0.0482 |
| C vs H | Steroid biosynthesis | ko00100 | 3 (6.122%) | 18 (0.422%) | 0.0010 |
|  | Nitrogen metabolism | ko00910 | 3 (6.122%) | 21 (0.492%) | 0.0016 |
|  | Alanine, aspartate and glutamate metabolism | ko00250 | 3 (6.122%) | 30 (0.704%) | 0.0047 |
|  | Drug metabolism - cytochrome P450 | ko00982 | 3 (6.122%) | 32 (0.750%) | 0.0056 |
|  | Progesterone-mediated oocyte maturation | ko04914 | 4 (8.163%) | 77 (1.806%) | 0.0113 |
|  | Linoleic acid metabolism | ko00591 | 2 (4.082%) | 22 (0.516%) | 0.0258 |
|  | Nicotinate and nicotinamide metabolism | ko00760 | 2 (4.082%) | 23 (0.539%) | 0.0281 |
|  | PPAR signaling pathway | ko03320 | 3 (6.122%) | 60 (1.407%) | 0.0309 |
|  | Amyotrophic lateral sclerosis (ALS) | ko05014 | 1 (2.041%) | 3 (0.070%) | 0.0341 |
|  | Antigen processing and presentation | ko04612 | 1 (2.041%) | 3 (0.070%) | 0.0341 |
|  | Retinol metabolism | ko00830 | 2 (4.082%) | 26 (0.610%) | 0.0353 |
|  | Metabolism of xenobiotics by cytochrome P450 | ko00980 | 2 (4.082%) | 31 (0.727%) | 0.0487 |
| M vs H | DNA replication | ko03030 | 6 (7.407%) | 31 (0.727%) | 0.0000 |
|  | Cell cycle | ko04110 | 8 (9.877%) | 111 (2.603%) | 0.0011 |
|  | Drug metabolism - cytochrome P450 | ko00982 | 4 (4.938%) | 32 (0.750%) | 0.0029 |
|  | Vitamin B6 metabolism | ko00750 | 2 (2.469%) | 8 (0.188%) | 0.0093 |
|  | Sulfur metabolism | ko00920 | 2 (2.469%) | 9 (0.211%) | 0.0118 |
|  | Folate biosynthesis | ko00790 | 2 (2.469%) | 10 (0.235%) | 0.0145 |
|  | Steroid hormone biosynthesis | ko00140 | 3 (3.704%) | 29 (0.680%) | 0.0169 |
|  | Metabolism of xenobiotics by cytochrome P450 | ko00980 | 3 (3.704%) | 31 (0.727%) | 0.0203 |
|  | Protein processing in endoplasmic reticulum | ko04141 | 7 (8.642%) | 158 (3.705%) | 0.0294 |
